# Comparative Study on the Flame-Retardant Properties and Mechanical Properties of PA66 with Different Dicyclohexyl Hypophosphite Acid Metal Salts

**DOI:** 10.3390/polym11121956

**Published:** 2019-11-28

**Authors:** Heng Zhang, Junliang Lu, Hongyan Yang, Jinyan Lang, Heng Yang

**Affiliations:** 1 Shandong Provincial Key Laboratory of biochemical engineering, College of Marine Science and Biological Engineering, Qingdao University of Science & Technology, Qingdao 266042, China; juling_lu@163.com (J.L.); y839461251@163.com (H.Y.); ljy17806248212@163.com (J.L.); kdjh401@163.com (H.Y.); 2 Key Laboratory of Biomass Chemical Engineering of Ministry of Education, Zhejiang University, Hangzhou 310027, China

**Keywords:** dicyclohexyl hypophosphite, PA66, flame-retardant properties, mechanical properties

## Abstract

Three metal salts of dicyclohexyl hypophosphite, namely dicyclohexyl aluminum hypophosphite (ADCP), dicyclohexyl magnesium hypophosphite (MDCP), and dicyclohexyl zinc hypophosphite (ZDCP), were synthesized. These flame retardants were subjected to thermogravimetric analysis, and the results showed that ADCP and ZDCP had higher thermal stabilities than MDCP. They were then separately mixed with polyamide 66 (PA66)to prepare composite materials, of which the combustion properties were determined by the limiting oxygen index method and horizontal/vertical burning experiments. The mechanical properties of the materials were further evaluated using an electronic universal testing machine. The results showed that all the three flame retardants exerted a flame-retardant effect on PA66, but the flame-retardant effect of MDCP was inferior to those of ADCP and ZDCP. All the composites also showed similar mechanical properties. Among the three flame retardants, ADCP had the best overall performance for raw materials, showing good flame-retardant properties while maintaining the mechanical properties of the raw materials. The optimal dosage of ADCP was 15 wt %, at which a V-0 rating in the vertical burning test (UL 94 test) can be obtained.

## 1. Introduction

Flame retardants have developed rapidly with extensive applications of polymer materials. Phosphorus-based flame retardants are an important class of nonhalogen flame retardants. They act as a flame retardant in gas and condensed phases but are more potent in the latter [1,2,3]. When a material is burned, a phosphorus-containing flame retardant is thermally decomposed into an oxo acid of phosphorus. The oxo acid of phosphorus can scavenge oxygen-containing groups produced by material burning and promote carbon layer formation. This carbon layer can protect the unburned polymer by preventing the cleavable product from being converted into a gaseous fuel and isolating it from heat, thereby inhibiting polymer combustion. Char formation is usually an endothermic reaction accompanied by the formation of water vapor, which is beneficial for lowering the ambient temperature and diluting the combustible gas.

The flame-retardant efficiency of a phosphorus-based flame retardant is related to the structure of the flame-retardant polymer and the molecular composition of the flame retardant. For polymers that do not contain oxygen in their molecular structures, such as styrene and polyolefins, phosphorus-based flame retardants have a poor char-forming effect. If no char-forming agent is added, the flame retardant will only be flame-retarded in the gas phase. The protective layer formed on the surface of the polymer is bound to have many droplets. Phosphorus-based flame retardants are classified into inorganic phosphorus flame retardants and organic phosphorus flame retardants according to their structures and compositions [4,5]. Among them, alkyl hypophosphite is a new organophosphorus flame retardant in the market. It exhibits the following excellent properties [6]: (1) good thermal stability and high flame-retardant efficiency; (2) good mechanical, electrical, and processing properties of products resulting from a combination with polymer materials; (3) good toxicological and environmental characteristics; and (4) halogen-free environmental protection.

Alkyl hypophosphite has the flame-retardant characteristics of phosphorous-based flame retardants. It can simultaneously exert a flame-retardant action effect in the condensed and gas phases. This flame retardant contains P–C, P–O, and P=O bonds, and its molecular formula is (R_2_POO)_n_^−^M_n_^+^, as shown in Formula (1) [6]:
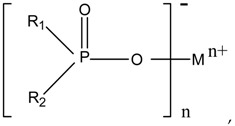
(1)
where R_1_ and R_2_ are C1–C6 alkyl (methyl, ethyl, *n*-propyl, isopropyl, *n*-butyl, *t*-butyl, and *n*-pentyl) and aryl (such as phenyl); M refers to a metal element, such as zinc, magnesium, and aluminum. Alkyl hypophosphite flame retardants are extensively studied, but researchers have primarily concentrated on aluminum ethyl hypophosphite and aluminum propyl hypophosphite. Germany Klein Company [7,8] synthesized aluminum diethylhypophosphite (AlPi) from sodium hypophosphite monohydrate and ethylene. After these materials were reacted at 100 °C for 6 h, additional aluminum hydroxide was added to continue the reaction for 2 h. The product was finally purified to provide a yield of 79.4%. To further optimize AlPi, Yang et al. [9] prepared a novel type of AlPi through a gas-liquid free-radical addition reaction under atmospheric pressure. This novel method increased the gas-liquid contact surface and the initiator concentration at the gas-liquid interface, thus increasing the yield of the final product to at least 80%. Braun et al. [10,11] investigated that glass-fiber-reinforced polybutylene terephthalate (PBT) with a 13–20 wt % loading range of aluminum diethylphosphinate fulfilled the flame-retardant requirements (a UL94 V-0 rating and a limiting oxygen index (LOI) value of >42%). Hu et al. [12] used the mixtures of aluminum salts of both diisobutylphosphinic acid and monoisobutylphosphinic acid (HPA–2TBA–Al) as flame retardants for glass-fibre-reinforced polyamide 6 (GFPA6). The GFPA6/25% HPA–2TBA–Al composite achieved a V-0 rating in the UL 94 test and had an increased LOI value of 34.0%. Tang et al. [13] found that aluminum hypophosphite (AHP) was fit to flame-retarded polylactide at a 20 wt % loading and the LOI value of composites was increased to 28.5%. Yuan et al. [14] studied the flame retardancy of aluminum dipropylphosphinate (ADPP) in polyamide 6 (PA6) by the thermogravimetric analysis (TGA), the LOI, and the UL 94 classification. They concluded that ADPP has good flame retardancy in nylon 6. Materials with 15 wt % ADPP achieve a V-0 rating in UL 94 tests and an increased LOI of 30.7%. Zhao et al. [15] added aluminum hypophosphite (AP) and aluminum isobutylphosphinate (APBu) to PA6. A novel binary flame-retardant system was formed by introducing AP and APBu together into PA6. The optimum flame-retardant formulation was 1:1 (AP:APBu; 15 wt % in total), and the resulting flame retardant PA6 could achieve an LOI of 28.3% and a UL 94 V-0 rating. Jian et al. [11] used aluminum isobutylphosphinate (APBu) and its synergistic system with red phosphorus (APBu/RP) to flame-retard acrylonitrile-butadiene-styrene (ABS). With the addition of APBu to ABS, the flame retardancy of the material was greatly improved, the LOI value was as high as 29.8%, and a UL 94 V-0 rating was obtained.

A new type of flame retardant, cyclohexyl hypophosphite, is less frequently studied. Zhang et al. [16,17,18] synthesized the dicyclohexyl aluminum hypophosphite (ADCP) flame retardant under normal pressure and compared its performance with that of diethyl aluminum hypophosphite (ADEP). Through LOI measurements and vertical burning tests, they found that the flame retardancy of ADCP was better than that of ADEP in polyamide. The optimal addition amount was 12%. At this time, the composite material had an LOI of 34.1% and a combustion rating of UL 94 V-0. They further explored the pyrolysis kinetics of the ADCP flame-retardant polyamide and the synergistic flame-retardant effect with nanosilica, and they found that the ADCP flame-retardant addition can improve the decomposition activation energy of polyamides, rendering the material difficult to burn. The addition of nanosilica can effectively improve the flame-retardant effect of ADCP. Compared with ethyl aluminum hypophosphite and propyl aluminum hypophosphite, ADCP can form a carbon foam layer together with PA66 on the surface of the material during combustion because of its high carbon content. This carbon layer acts as a heat insulator, an oxygen insulator, a smoke suppressor, and a drip preventer. ADCP can also simultaneously allow for excellent flame-retardant performance of the gas-phase and condensed carbon layer. The system itself has a synergistic flame-retardant effect. It is a new generation of halogen-free organic phosphorus flame-retardant material.

The effects of the alkyl group type on the alkyl aluminum hypophosphite flame retardants have been studied. However, the effects of different metal salts, especially commonly used aluminum, magnesium, and zinc salts, on alkyl hypophosphite have not been reported. In the present work, three high-yield dicyclohexyl hypophosphite flame retardants were prepared and separately added to PA66. The flame retardancy and the combustion properties of the resulting composite materials were analyzed by LOI measurements and vertical combustion experiments, respectively. Their mechanical properties (tensile and bending strengths) were measured using an electronic universal testing machine. The suitability of the three flame retardants to the materials was evaluated.

## 2. Materials and Methods

### 2.1. Experiment Reagents

PA66 particles (industrial grade) were obtained from DuPont Corporation. Sodium hypophosphite (with analytical purity) was purchased from Shanghai Aibe Chemical Reagent Co., Ltd (Shanghai, China). Glacial acetic acid, cyclohexene, benzoyl peroxide, sodium hydroxide, zinc sulfate, sulfuric acid, aluminum sulfate, and magnesium sulfate (with analytical purity) were acquired from Sinopharm Chemical Reagent Co., Ltd (Shanghai, China).

### 2.2. Sample Preparation

#### 2.2.1. Preparation of Dicyclohexyl Hypophosphite

**Figure 1:**
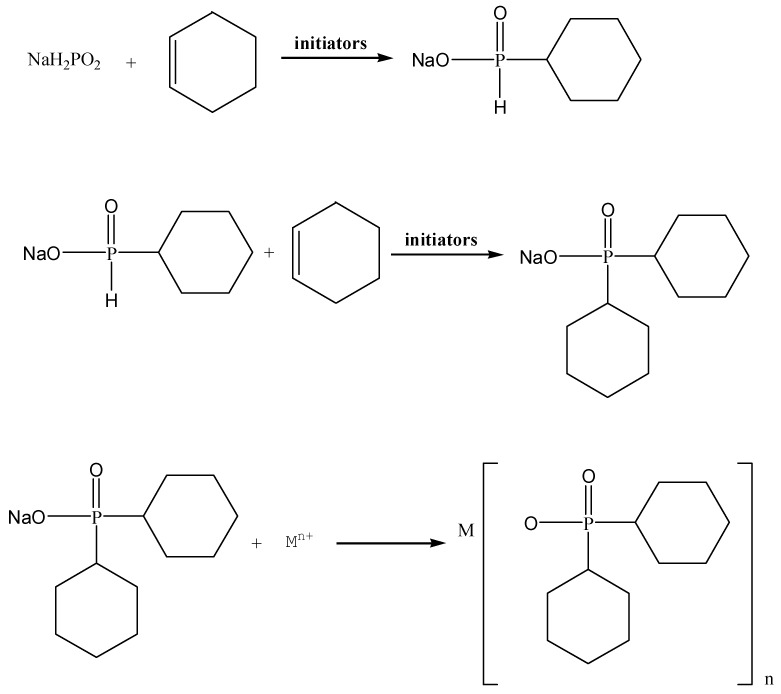
Synthesis route of dicyclohexyl hypophosphite. M^n+^ is Al^3+^, Mg^2+^, or Zn^2+^.

In a 250 mL four-neck flask, 10.6 g (0.1 mol) of sodium hypophosphite was dissolved in 30 mL of glacial acetic acid, and a suitable agitator was assembled. Subsequently, 24.65 g (0.3 mol) of cyclohexene was weighed and added to the four-neck flask. A condensation tube and a thermometer were installed in the four-neck flask, nitrogen was passed, and temperature was increased to 85 °C. Finally, benzoyl peroxide dissolved in glacial acetic acid was added dropwise into the flask. The reaction temperature and time were controlled until the end of the reaction, and the product was cooled to room temperature. The product was removed by vacuum distillation to obtain pure dicyclohexyl sodium hypophosphite. An aqueous solution of dicyclohexyl sodium hypophosphite was prepared by adding deionized water, and a NaOH solution was added to adjust the pH to obtain an alkaline dicyclohexyl sodium hypophosphite solution. The solution was poured into a 500 mL four-neck flask, and an appropriate blender and a reflux condensation tube were assembled. A certain concentration of a metal salt aqueous solution was added dropwise to the solution and the reaction temperature and time were controlled. The solid precipitate was obtained by neutralizing the solution with 30% sulfuric acid. After it was cooled to room temperature, the solid was washed with deionized water and centrifuged three times. The dicyclohexyl hypophosphite solid was obtained after drying [16,17]. The synthetic route is shown in Figure 1.

#### 2.2.2. Preparation of the Flame Retardant-PA66 Composite Materials

PA66 particles were dried for 10 h in a vacuum drying chamber (DZF-6020, Shanghai Yiheng Scientific Instruments Co., Ltd, Shanghai, China) at 80 °C to remove excess water. The PA66 particles were melted in a 250 °C mixer (SU-70B, Changzhou Suyan Technology Co., Ltd, Changzhou, China) and then blended with the flame retardants of different qualities for 10 min to allow the flame retardants to be evenly dispersed throughout the matrix. Finally, the mixture material was added to the mold, and a polymer calendar sheet was pressed with a high-precision automatic tablet press (2G-10T, Dongguan Zhenggong Mechanical and Electrical Equipment Technology Co., Ltd, Dongguan, China) at 260 °C and 10 MPa.

### 2.3. Thermogravimetric Analysis of Dicyclohexyl Hypophosphite

The product was examined by thermogravimetric analysis with the DT-50 thermogravimetric analyzer (Setaram Instrumentation, Lyons, France) in nitrogen and air atmosphere. Approximately 10 mg of samples was placed in an alumina crucible and tested with a thermogravimetric analyzer, of which the temperature was increased from 0 to 800 °C. The heating rate was 20 °C/min. Both the nitrogen and air flow rates were 20 mL/min.

### 2.4. Flame-Retardant Property Test of Dicyclohexyl Hypophosphite on PA66

The LOI was measured with the JF-3 oxygen index analyzer (Nanjing Jiangning District Analytical Instrument Factory, Nanjing, China). The polymer sheets were cut into splines of certain specifications by using a woodworking band saw machine. According to the standard GB/T 2406.1-2008 shown in *Measuring Combustion Behavior of Plastics by Oxygen Index Method*, the spline size was 120 mm × 6.5 mm × 3.0 mm [19,20,21]. The CZF-3 horizontal vertical burning tester (Nanjing Jiangning District Analytical Instrument Factory, Nanjing, China) was then used for vertical combustion experiments. Splines were tested in accordance with the national standard GB/T 2408-2008 [22].

### 2.5. Mechanical Property Test of Dicyclohexyl Hypophosphite on PA66

The tensile and flexural strengths of the samples were tested with the UTM2502 electronic universal testing machine (Jinan Kesheng Test Equipment Co., Ltd, Jinan, China). The tensile strength was measured according to the standard GB/T 1040.1-2006 shown in *Testing of Tensile Properties of Plastics*. The flexural strength was measured according to the standard GB/T 9341-2008 shown in *Testing of Bending Properties of Plastics* [23,24]. The test speed was 2 mm/min.

## 3. Results and Discussion

### 3.1. Flame-Retardant and Mechanical Properties of the PA66/ADPC Composites

ADPC was added to PA66 in various proportions, and the flame-retardant properties are shown in Table 1.

Table 1 shows that the addition of ADCP to PA66 greatly influenced the flame retardancy of PA66. The pure PA66 burnt violently in fire. The UL 94 test had a V-2 rating, and the LOI was 21.5%. The experiment was accompanied by severe droplet dripping. After adding the ADCP flame retardant, the LOI of the composites were significantly improved, and the value increased with the increased flame retardant addition in a certain range. This result indicates that the ADCP addition can significantly increase the flame retardant properties of PA66. When 15 wt % ADCP was added, the LOI of the composite material was 32%, which was 48.8% higher than that of the pure PA66. The above conclusion was well confirmed by the cone calorimetry test and EDS test, and the SEM images showed the formation of homogeneous carbon layers (please see Supplementary Figures S1–S4 and Table S1). The increase ratio was 48.8%. The UL 94 test of the composite material had a V-0 rating, and no liquid droplet dripping was observed. With the further increased addition of the flame retardant, its LOI did not considerably change. Thus, the optimal dosage of ADCP was 15 wt %.

**Figure 2:**
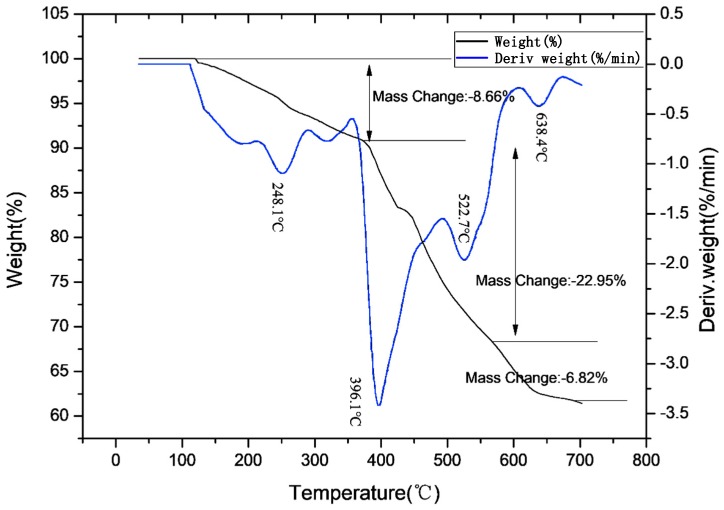
Thermogravimetric curve of ADCP in N_2_ atmosphere.

**Figure 3:**
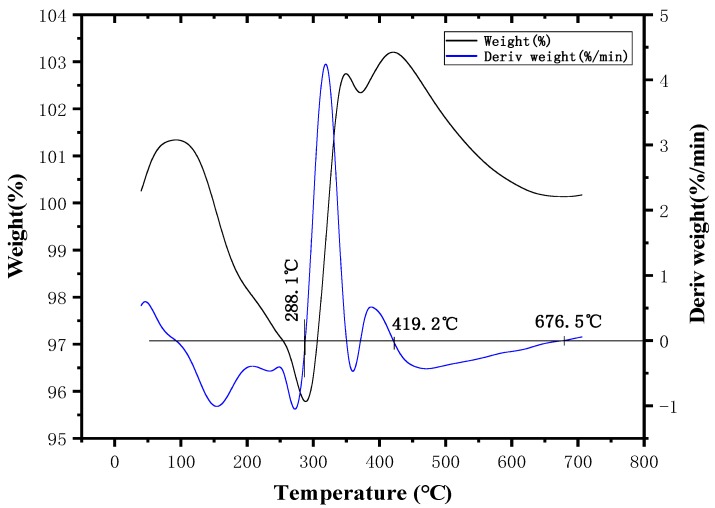
Thermogravimetric curve of ADCP in air atmosphere.

Figure 2 shows that the ADCP had three weight loss intervals in N_2_ atmosphere and the starting weight loss temperature was 110 °C. The first weight loss interval temperature was 110–350 °C, which was primarily due to the removal of a small amount of water and small molecular substances. Based on the mechanism of product synthesis, the authors believed that these small molecules were derived from hypophosphite, monocyclohexyl hypophosphite, and their thermally decomposed products that were not completely removed. The structural stabilities of these small molecules were weaker than that of ADCP with two cyclohexyl groups, and these molecules thus decomposed in advance. Among them, these small molecules with hypophosphite groups had a flame-retardant effect. The temperature of the second weight loss interval was 350–562 °C, which was primarily due to dehydration and carbonization of anionic groups by thermal decomposition. In this interval, the metal salt got rid of ionic bonding. The anionic group decomposition of the product began to exert a flame-retardant effect and form a carbon layer. While the composite was formed with PA66 and burned, the phosphorus group decomposed, capturing the active groups generated by combustion in the gas phase and the solid phase, and the cyclohexyl group of carbonization participated in the formation of the carbon layer of PA66. The temperature of the third weight loss interval was 562–610 °C, because the carbon layer formed in the previous interval was decomposed at high temperatures. With the further increased temperature, the weight of the ADCP remained almost the same, at which point the flame retardant broke down into stable small molecules. When the composite of ADCP and PA66 was burned, these small molecules were used as fillers to fill the carbon layer formed by the composite combustion, which increased the compactness of the carbon layer and improved the flame retardant effect. As shown in Figure 3, the curve started to rise at 300 °C. The main reason was that oxygen in the air reacted with ADCP to form stable solid compounds. The introduction of these oxygen atoms macroscopically affected the weight loss of ADCP on the thermogravigram. Additionally, the flame-retardant mechanism did not change at a microscopic level. In conclusion, ADCP has a good thermal stability and can be added as a flame retardant to polymer materials.

As shown in Table 2, the tensile strength of the material decreased, and the bending strength increased with the addition of ADCP in PA66. This result proves that the addition of ADCP affects the mechanical properties of PA66. The main reason for the decreased tensile strength is that ADCP replaced part of the PA66 component in the material. When the amount of ADCP added was 15 wt %, the tensile strength of the composite material was 49.6 MPa, and the bending strength was 55.4 MPa. Compared with the pure sample, the composite material shows a 12.1% decrease in tensile strength and a 3.8% increase in bending strength. Although this ratio had a certain influence on the mechanical properties of PA66, the mechanical properties of PA66 are generally maintained at this ratio, while the good flame-retardant properties are maintained.

### 3.2. Flame-Retardant and Mechanical Properties of the PA66/MDCP Composites

Experiments were performed by proportionally adding MDCP into PA66. The results are shown in Table 3.

The test results in Table 3 show that the MDCP addition did not significantly influence the flame retardancy of PA66. Severe melt dripping occurred during the experiment. The LOI of the samples minimally increased with the increased flame retardant volume. When 25% of MDCP was added, the LOI of the composite material was only 25%, The UL 94 test had a V-2 rating, and the melt dripping still occurred. This finding indicates that MDCP does not improve the flame-retardant property of PA66 and is thus unsuitable for this type of material.

**Figure 4:**
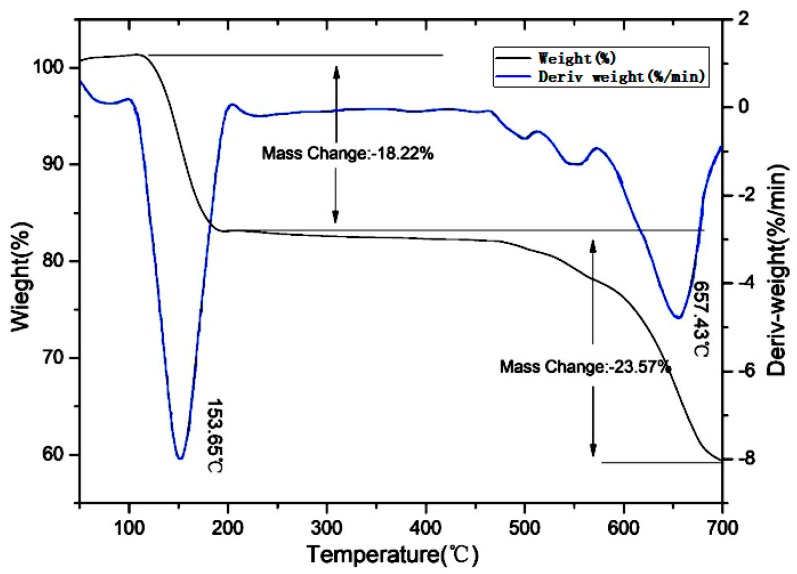
Thermogravimetric curve of MDCP in N_2_ atmosphere.

**Figure 5:**
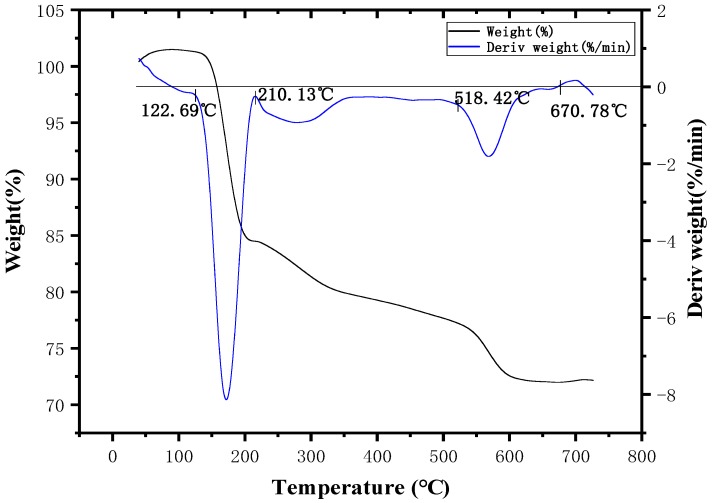
Thermogravimetric curve of MDCP in air atmosphere.

Figure 4 shows that MDCP had two weight loss intervals starting at 104 °C. The first weight loss occurred at 104–196 °C, which was primarily due to the removal of a small amount of water and small molecular substances as well as the dehydration and the carbonization of part of anionic groups caused by thermal decomposition. The thermal decompositions of the components are similar to that of ADCP. The second weight loss occurred at 485–685 °C. In this interval, a large amount of the anionic groups were dehydrated and carbonized, and the formed carbon layer was decomposed with the increase of temperature. The anion groups of the three flame retardants were identical, so the decomposition principle of this interval is the same as that of ADCP. The three weight loss intervals can be seen with thermogravimetry under air (Figure 5). It can be seen that the anionic group of the flame retardant decomposed at 200–500 °C, shown in Figure 4. However, magnesium and nitrogen can generate magnesium nitride at high temperatures, so the weight loss of MDCP was not obvious at the interval. At 700 °C, MDCP still had a mass surplus of 59.75%. Hence, MDCP cannot meet the required processing temperature of polymer materials due to the common heat instability and the decomposition reaction occurring below 350 °C. These findings show that this flame retardant cannot be applied to PA66.

The data in Table 4 show that the tensile strength decreased whereas bending strength increased with the increased MDCP content in PA66. The main reason for the decreased tensile strength is the same as previously discussed. Compared with PA66 of the same purity, the composite added with 25% of MDCP shows a 29.9% decrease in tensile strength by and a 9% increase in bending strength. This result reveals that MDCP influences the mechanical properties of PA66.

### 3.3. Flame-Retardant and Mechanical Properties of the PA66/ZDCP Composites

ZDCP was added to PA66 in various proportions, and the flame-retardant performance results are shown in Table 5.

Table 5 illustrates that the addition of ZDCP to PA66 improves its LOI. ZDCP greatly affected the vertical horizontal combustion, which is manifested as the disappearance of a drop of molten liquid. When the ZDCP flame retardant was added, the vertical horizontal combustion was accompanied by serious dripping of molten liquid and reburning, and the drop of molten liquid gradually disappeared with further addition of the flame retardant. When 15 wt % ZDCP was added, the LOI of the composite was 26.5%, and the UL 94 test had a V-1 rating with dripping of the molten liquid. When 25% ZDCP was added, the LOI of the composite was 30.5%, and the UL 94 test had a V-0 rating without dripping of the molten liquid. Thus, ZDCP can improve the LOI of the PA66 composite, the vertical combustion performance, and the flame-retardant effect.

**Figure 6:**
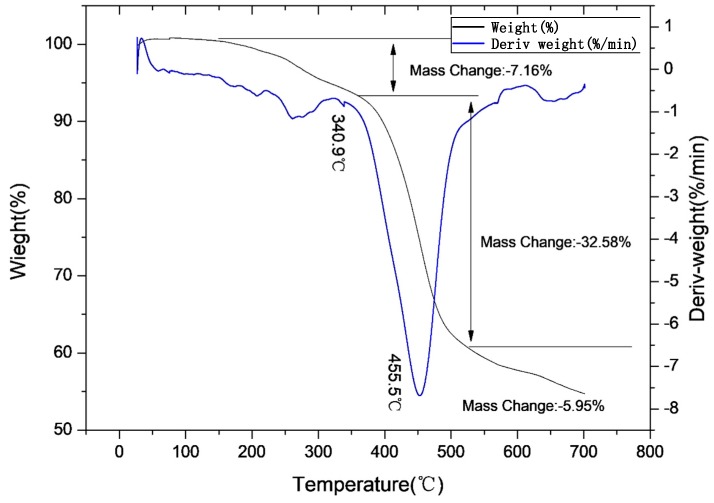
Thermogravimetric curve of ZDCP in N_2_ atmosphere.

**Figure 7:**
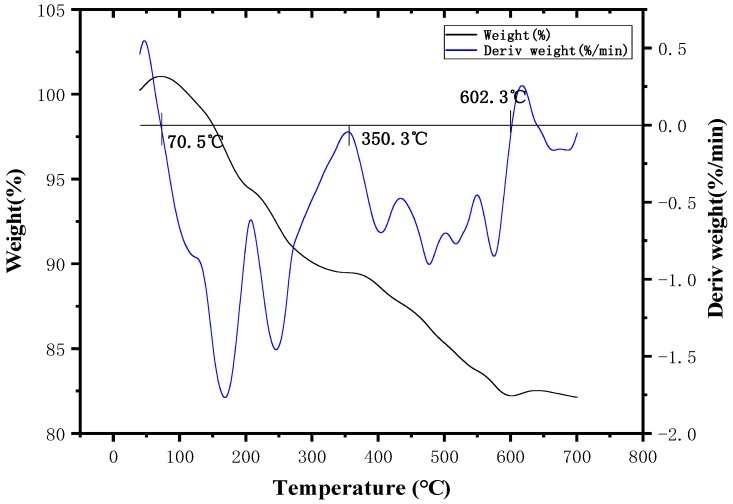
Thermogravimetric curve of ZDCP in air atmosphere.

Figure 6 shows that ZDCP had three weight loss intervals in N_2_ atmosphere. The temperature of the first weight loss interval was 40–340.9 °C, which was primarily due to the removal of a small amount of water and small molecular substances. These small molecular components are similar to the initial thermal-decomposition components of ADCP. The temperature of the second weight loss interval was 340.9–533.2 °C, which was primarily due to the dehydration and the carbonization of the anionic groups. It indicates that the product has good carbonization performance while being flame-retardant. The decomposition principle is the same as that of ADCP. The temperature of the third weight loss interval was 533.2–610 °C, and the carbon layer decomposition occurred in this interval. Beyond 700 °C, there was still a mass surplus of 54.31%. At temperatures of <340.9 °C, ZDCP did not easily decompose and lose weight, indicating the good thermal stability of the product. Thus, it can meet the processing temperature requirements of engineering plastics. Compared with Figure 6 and Figure 7, the weight loss of ZDCP in air was significantly lower than that in N_2_. The reason is the same as for ADCP. However, ADCP is superior to ZDCP in flame retardancy due to the different oxygen contents in the air around the flame retardant after the formation of the metal oxide.

As shown in Table 6, with the increased ZDCP in PA66, the tensile strength of the material decreased, and the bending strength increased. This result indicates that the ZDCP addition affected the mechanical properties of PA66. The main reason for the decreased tensile strength is the same as previously discussed. Compared with the pure PA66, PA66 with 25% ZDCP shows a 28.5% decrease in tensile strength and a 10.3% increase in bending strength, indicating that ZDCP maintains good flame retardancy of PA66 and its mechanical properties. The experimental results are similar to those observed in the ADCP/PA66 composites.

Based on the above experimental results, the flame retardancy of dicyclohexyl hypophosphite is directly related to the metal species it carries. ADCP and ZDCP have good flame-retardant effects on PA66, whereas the flame-retardant effect of MDCP is not evident. This condition may be due to the thermal stability of different metal ions differing in the salt-formation process and the amount of charge carried by these ions themselves. On the one hand, the thermogravimetric analysis shows that the Gibbs free energy change (∆*G*) of the three metal ion-prepared flame retardants differed when they were thermally decomposed. A larger ∆G means greater thermodynamic stability of the flame retardant. It macroscopically caused the carbonization temperature and the carbon oxidation decomposition temperature of the flame retardant itself to be different. The carbonization temperatures of ADCP, ZDCP, and MDCP were 350, 340.9, and 104 °C in N_2_, respectively. Given that the melting temperature range of PA66 particles was from 104 to 340 °C, most of the MDCP was added as a carbonized product during the preparation of the composite material, affecting the structure of the carbon layer on the surface of the material. When the flame retardant and PA66 were mixed in a molten state, the flame retardant was relatively uniformly distributed throughout PA66 due to molecular thermal motion. When the composite was burned, the flame retardant itself carbonized and promoted the PA66 carbonization. Although the carbon formed by the two participates in the carbon layer formation on the surface of the material, the difference in the structure of the two carbons resulted in different oxidative decomposition temperatures. Among the three flame retardants, MDCP has the lowest carbon oxidation decomposition temperature (485 °C). During the combustion, the carbon layer formed by MDCP was initially destroyed, changing the overall compactness of the carbon layer. Thus, the carbon layer’s heat insulation effect worsened, and the flame-retardant effect was reduced. ADCP (562 °C) had a higher carbon oxidative decomposition temperature than ZDCP (533.2 °C). Therefore, MDCP has the poorest flame-retardant effect, and ADCP has a better flame-retardant effect than ZDCP.

On the other hand, the three metal ions have different numbers of phosphate groups in their respective flame retardants. Aluminum ions have one more electrons than zinc and magnesium ions. Thus, one additional phosphorus group is involved in salt formation. The data in Table 7 were obtained by calculations. The coefficients of 1.369 (ZDCP coefficient) and 1.481 (MDCP coefficient) were introduced to make the relative molecular masses of ZDCP and MDCP the same as that of ADCP, respectively. Both the sets of coefficients are in the range of (1,1.5). When the same mass fraction of flame retardant was added, the molar numbers of the phosphorus-containing groups in the three flame retardants are arranged in descending order as follows: ADCP > MDCP > ZDCP. These phosphorus-containing groups have strong dehydration carbonization, and ADCP has more phosphate groups. Thus, ADCP can accelerate the formation of the carbon layer and the combustion material carbon layer during combustion and capture more active radicals in the gas phase. Therefore, ADCP has the best flame-retardant effect [13].

However, these flame retardants have the same tendency to change the mechanical properties of the original materials, i.e., reduction of their tensile strengths and increase of their bending strengths. This finding is due to the addition of the dicyclohexyl hypophosphite flame retardant replacing part of PA66. The addition of the new material changed the single-molecule force between the PA66 molecules. At this time, the intermolecular forces of the composites were between the PA66 molecules, between the flame retardant and PA66, and between the flame retardant molecules. Given that polyamide is a polymer with a polar amide group (–CO–NH–) in the main chain link, the addition of the dicyclohexyl hypophosphite flame retardant can form a hydrogen bond with the polyamide molecule. Hydrogen bonds with bond energies weaker than those of covalent bonds replaced some covalent bonds, which weakened the overall bond energy of the composite materials. As a result, the bond energy of the new material at the particle points with the flame retardants was low, and the material had some weak tensile stress points, which led to the decrease of the tensile strength of the composite material.

In addition, the large spatial steric resistance of the flame retardants changed the regular arrangement structure of the raw materials to some extent and changed the force uniformity locally. From the microscopic point of view, since both the covalent bonds and the hydrogen bonds had directivity, the generation of hydrogen bonds caused local changes in the direction of the intermolecular forces in materials. At this point, the material had stress points in both vertical and horizontal directions, which made the material have better toughness when bending. Therefore, the bending strength of the material was increased.

## 4. Conclusions

Adding the single-component flame retardants, namely ADCP, MDCP, and ZDCP, to PA66 reduced the tensile properties of the composite but increased its flexural strength. In terms of the flame retardancy, the LOI of the three flame retardants increased within a certain range with the increased amount of the flame retardants. The LOI tended to be stable with the increased amount of the flame retardants to a certain extent. Although these flame retardants all had flame-retardant effects, the flame-retardant effects of ADCP and ZDCP were significantly higher than that of MDCP, and that of ADCP was the best. When 15 wt % ADCP was added, the LOI of the composite tended to be stable, no melt dripping occurred during the combustion, and the UL 94 test had a V-0 combustion rating. The same degree of ZDCP had a relatively low flame-retardant effect. Its combustion rating was V-1, and the melting dropped drips during the combustion. The MDCP had the poorest flame-retardant effect. The LOI of the composite material was not greatly improved throughout the entire test process and was accompanied by severe melting drops dripping during the combustion. Thus, MDCP cannot be applied in PA66.

## Figures and Tables

**Table 1:** Formulation and flame retardancy of PA66 and its composites.

Sample	PA66	Dicyclohexyl Aluminum Hypophosphite (ADCP)	Limiting Oxygen Index (LOI)	UL 94	
	(wt %)	(wt %)	(%)	Dripping	Rating
PA66-0	100	0	21.5 ± 0.9	Y	V-2
PA66-1	95	5	24.0 ± 0.7	Y	V-1
PA66-2	90	10	28.0 ± 0.5	N	V-0
PA66-3	85	15	32.0 ± 0.9	N	V-0
PA66-4	80	20	33.1 ± 0.7	N	V-0
PA66-5	75	25	34.1 ± 0.8	N	V-0

**Table 2:** Effects of different amounts of added ADCP on the mechanical properties of the composites.

Sample	Mechanical Properties	
	Tensile Strength (MPa)	Bending Strength (MPa)
PA66-0	56.4 ± 0.6	53.4 ± 0.9
PA66-1	55.2 ± 0.9	53.4 ± 0.5
PA66-2	52.8 ± 0.7	54.1 ± 0.6
PA66-3	49.6 ± 0.6	55.4 ± 0.8
PA66-4	45.4 ± 0.5	56.8 ± 1.0
PA66-5	40.5 ± 0.7	57.8 ± 0.8

**Table 3:** Formulation and flame retardancy of PA66 and its composites.

Sample	PA66	Dicyclohexyl Magnesium Hypophosphite (MDCP)	LOI	UL 94	
	(wt %)	(wt %)	(%)	Dripping	Rating
PA66-0	100	0	21.5 ± 0.8	Y	Burning
PA66-1	95	5	22.0 ± 0.9	Y	Burning
PA66-2	90	10	23.0 ± 0.6	Y	Burning
PA66-3	85	15	23.5 ± 0.5	Y	V-2
PA66-4	80	20	25.0 ± 0.8	Y	V-2
PA66-5	75	25	25.0 ± 0.7	Y	V-2

**Table 4:** Effects of different amounts of added MDCP on the mechanical properties of the composites.

Sample	Mechanical Properties	
	Tensile Strength (MPa)	Bending Strength (MPa)
PA66-0	56.4 ± 0.6	53.4 ± 0.8
PA66-1	52.2 ± 0.8	53.8 ± 0.9
PA66-2	50.8 ± 0.9	54.9 ± 0.7
PA66-3	47.6 ± 0.7	55.9 ± 1.1
PA66-4	44.2 ± 1.1	57.0 ± 0.9
PA66-5	39.5 ± 0.9	58.2 ± 0.8

**Table 5:** Formulation and flame retardancy of PA66 and its composites.

Sample	PA66	Dicyclohexyl Zinc Hypophosphite (ZDCP)	LOI	UL 94	
	(wt %)	(wt %)	(%)	Dripping	Rating
PA66-0	100	0	21.5 ± 0.7	Y	Burning
PA66-1	95	5	23.0 ± 0.9	Y	Burning
PA66-2	90	10	24.5 ± 0.8	Y	V-2
PA66-3	85	15	26.5 ± 0.9	Y	V-1
PA66-4	80	20	29.0 ± 0.9	N	V-0
PA66-5	75	25	30.5 ± 0.7	N	V-0

**Table 6:** Effects of different amounts of added ZDCP on the mechanical properties of the composites.

Sample	Mechanical Properties	
	Tensile Strength (MPa)	Bending Strength (MPa)
PA66-0	56.4 ± 0.7	53.4 ± 0.9
PA66-1	53.2 ± 0.6	53.6 ± 0.7
PA66-2	50.8 ± 0.9	54.7 ± 0.6
PA66-3	47.2 ± 0.9	55.9 ± 0.8
PA66-4	44.7 ± 0.6	57.6 ± 0.9
PA66-5	40.3 ± 0.8	58.9 ± 0.7

**Table 7:** Relative molecular mass, number of moles, and number of moles of anion of the three flame retardants.

Flame Retardant	Relative Molecular Mass (g/mol)	Number of Phosphorus Groups with the Same Mass M (mol)
ADCP	745.062	3 M/745.062
ZDCP	544.110	2 M/544.110
MDCP	503.025	2 M/503.025

## Supplementary Materials

The following are available online at https://www.mdpi.com/2073-4360/11/12/1956/s1, Figure S1: Heat release rate (HRR) curves of PA66 and the flame retardant-PA66 (FR-PA66). 1, PA66; 2, 15% ADCP/PA66, Figure S2: Total heat release (THR) curves of PA66 and the FR-PA66. 1, PA66; 2, 15% ADCP/PA66, Figure S3: SEM images of the carbon layer after a cone calorimetry test: (A) the pure sample of PA66; (B) the 15% ADCP/PA66 composite, Figure S4: Various element contents and energy dispersive spectroscopy (EDS) results of the products: (a) surface of the carbon layer; (b) section of the carbon layer, Table S1: Peak of mass loss rate (MLR) and peak of smoke production rate (SPR) of PA66 and its composites.
