# Supplementary material for: Comparative Study on the Flame-Retardant Properties and Mechanical Properties of PA66 with Different Dicyclohexyl Hypophosphite Acid Metal Salts
Source: Polymers (Basel). 2019 Nov 28;11(12):1956. doi: 10.3390/polym11121956 (PMC6960571; doi:10.3390/polym11121956)
Supplement: Supplementary file 1 [file polymers-11-01956-s001.pdf]

polymers

Supplementary material

Comparative Study on the Flame-Retardant Properties and Mechanical Properties of PA66 with Different Dicyclohexyl Hypophosphite Acid Metal Salts

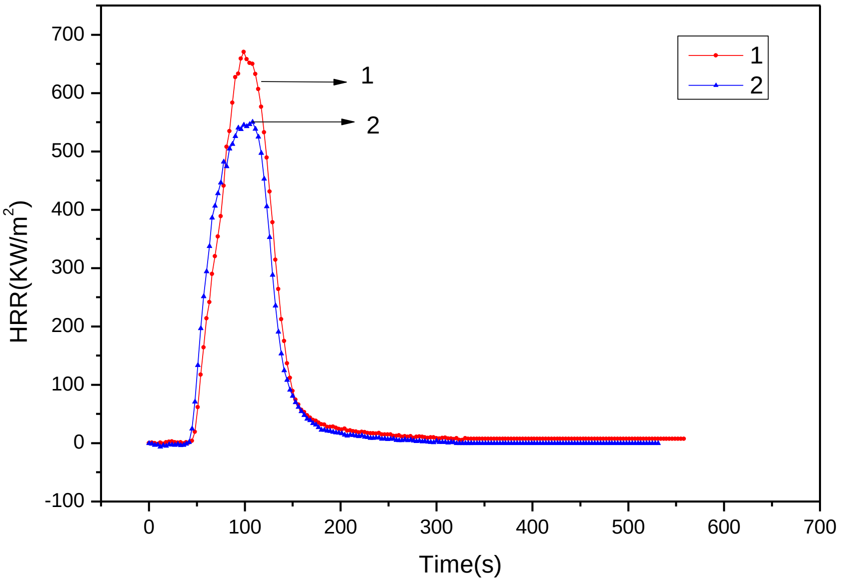

Figure S1. Heat release rate (HRR) curves of PA66 and the flame retardant-PA66 (FR-PA66). 1, PA66; 2, 15% ADCP/PA66.

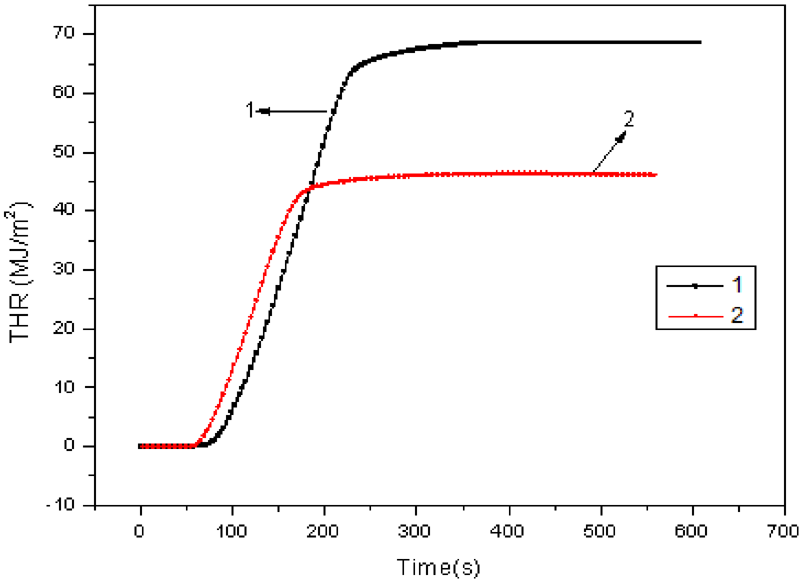

Figure S2. Total heat release (THR) curves of PA66 and the FR-PA66. 1, PA66; 2, 15% ADCP/PA66.

Figures S1 and S2 show the HRR and the THR data of the pure PA66 and the 15% ADCP/PA66 at a 35 kW/m^2 thermal radiation power. The HRR of the 15% ADCP/PA66 composite decreased, with a peak value and a total HRR of 546.5 KW/m^2 and 46.3 MJ/m^2, respectively. Therefore, the flame-retardant ADCP could substantially reduce the HRR and THR rates of the PA66. This effect was caused by the dicyclohexenyl aluminum hypophosphite in the condensed phase. The formed carbon layer prevented heat, oxygen, and flammable gases from entering the flame zone. In addition, it prevented the flame development and further combustion, and reduced heat release, thus achieving a flame-retardant effect.

Table S1. Peak of mass loss rate (MLR) and peak of smoke production rate (SPR) of PA66 and its composites.

	**PA66**	**15% ADCP/PA66 Composite**
Peak MLR(g/s)	0.13	0.08
Peak SPR(m^2/s)	0.16	0.12

Table S1 shows the peak of MLR and the peak of SPR of PA66 and its composites. As shown in Table S1, the peak of MLR of the 15% ADCP/PA66 was substantially lower than that of the pure PA66 sample. This finding showed that ADCP promoted char formation. In terms of SPR, the addition of the ADCP flame retardant substantially reduced the peak of SPR of the PA66 composites.

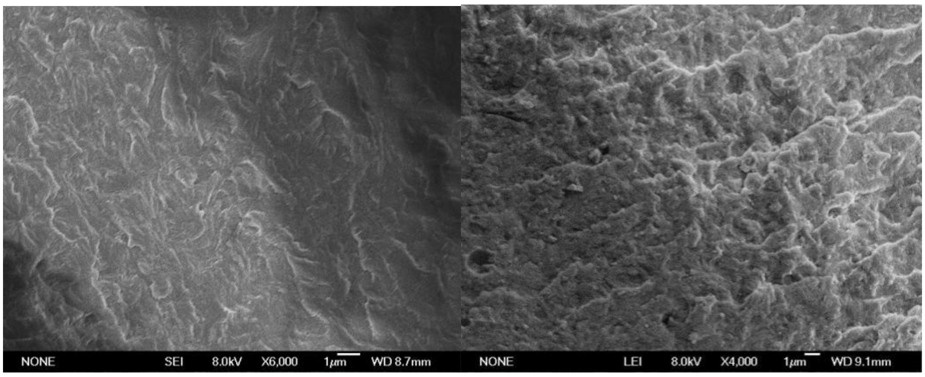

（**A**） （**B**）

Figure S3. SEM images of the carbon layer after a cone calorimetry test: (**A**) the pure sample of PA66; (**B**) the 15% ADCP/PA66 composite.

In contrast to the smooth surface in (**A**), the uneven surface in (B) is obviously rough, which represented the carbon layer formed during the combustion process of the flame retardant. All the parts in the images have the same morphology, indicating the uniform distribution of the flame retardants.

**Element**	**Weight (%)**	**Atomic (%)**
C	77.47	83.58
O	17.52	14.19
Al	0.39	0.19
Si	2.62	1.21
P	1.99	0.83
Totals	100.00	

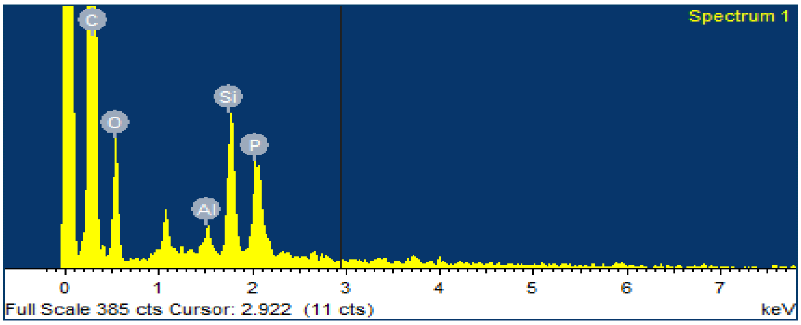

(**a**)

**Element**	**Weight (%)**	**Atomic (%)**
C	70.83	77.58
O	24.94	20.50
Al	0.74	0.36
Si	1.61	0.76
P	1.88	0.80
Totals	100.00	

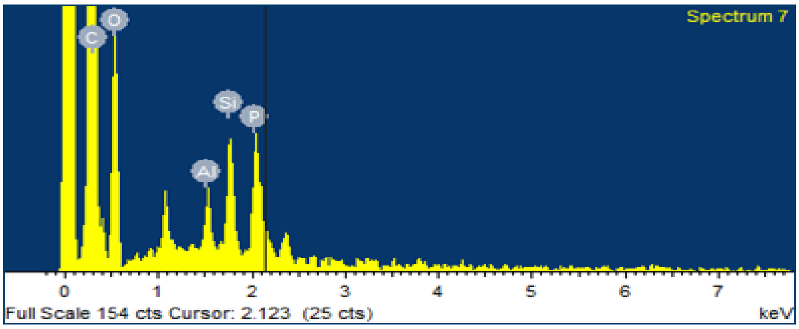

(**b**)

Figure S4. Various element contents and energy dispersive spectroscopy (EDS) results of products: (**a**) surface of the carbon layer; (**b**) section of the carbon layer.

Figure S4 shows the energy dispersive spectroscopy (EDS) analyses of the surface and the cross-section of the residue after a cone calorimetry experiment. The EDS analysis showed that the carbon content on the surface of the carbon layer was higher than that on the cross-section because of the combustion and the carbonization on the surface of the material. The phosphorus content was similar in both sections. This indicates that the flame retardants were evenly distributed in PA66.
